# Hospital mortality and length of stay differences in emergency medical admissions related to ‘on-call’ specialty

**DOI:** 10.1007/s11845-022-03084-w

**Published:** 2022-07-08

**Authors:** Richard P. Conway, Declan G. Byrne, Deirdre M. R. O’Riordan, Bernard Silke

**Affiliations:** 1 Department of Internal Medicine, St. James’s Hospital, Dublin 8, Ireland; 2 Clinical Medicine, Trinity College Dublin, Dublin, Ireland

**Keywords:** Acute medicine, Admissions, Length of stay, Mortality, Speciality

## Abstract

**Background:**

The outcomes of acute medical admissions have been shown to be influenced by a variety of factors including system, patient, societal, and physician-specific differences.

**Aim:**

To evaluate the influence of on-call specialty on outcomes in acute medical admissions.

**Methods:**

All acute medical admissions to our institution from 2015 to 2020 were evaluated. Admissions were grouped based on admitting specialty. Thirty-day in-hospital mortality and length of stay (LOS) were evaluated. Data was analysed using multivariable logistic regression and truncated Poisson regression modelling.

**Results:**

There were 50,347 admissions in 30,228 patients. The majority of admissions were under Acute Medicine (47.0%), and major medical subspecialties (36.1%); Elderly Care admitted 12.1%. Acute Medicine admissions were older at 72.9 years (IQR 57.0, 82.9) vs. 67.2 years (IQR 50.1, 80.2), had higher Acute Illness Severity (grades 4–6: 85.9% vs. 81.3%; *p* < 0.001), Charlson Index (> group 0; 61.5% vs. 54.6%; *p* < 0.001), and Comorbidity Score (40.7% vs. 36.7%; *p* < 0.001). Over time, there was a small (+ 8%) but significant increase in 30-day in-hospital mortality. Mortality rates for Acute Medicine, major medical specialties, and Elderly Care were not different at 5.1% (95% CI: 4.7, 5.5), 4.7% (95% CI: 4.3, 5.1), and 4.7% (95% CI: 3.9, 5.4), respectively. Elderly Care admissions had shorter LOS (7.8 days (95% CI: 7.6, 8.0)) compared with either Acute Medicine (8.7 days (95% CI: 8.6, 8.8)) or major medical specialties (8.7 days (95% CI: 8.6, 8.9)).

**Conclusion:**

No difference in mortality and minor differences in LOS were observed. The prior pattern of improved outcomes year on year for emergency medical admissions appears ended.

## Introduction

Acute Medicine relates to the immediate and specialist care and management of patients presenting to an acute hospital requiring urgent care for a broad spectrum of conditions. The process of care delivery has been shown to influence outcomes, most notably with significantly improved outcomes demonstrated following the introduction of an Acute Medical Assessment Unit (AMAU) [1, 2].

A variety of other factors have been shown to influence or predict outcomes, these include patient-specific factors such as illness severity and comorbidity and societal factors such as deprivation, day of admission, and laboratory tests [3–9]. Practice characteristics of the treating consultant physician such as experience and patient volume also appear to mediate outcomes [10, 11].

Knowledge of the factors influencing outcomes in acute medical admissions is vital for system planning, resourcing, prognostication, and most importantly targeting improved outcomes.

In this paper, we sought to assess the influence of the specialty of the admitting team and consultant on outcomes in acute medical admissions.

## Methods

### Background

St. James’s Hospital, Dublin, serves as a secondary care centre for emergency admissions in a catchment area with a population of 270,000 adults. St. James’s is based in an inner-city area with an aging population and high intrinsic rates of deprivation and comorbidity [12, 13]. All emergency medical admissions are admitted from the Emergency Department (ED) to an AMAU, the operation and outcome of which have been described elsewhere [1, 2, 14].

### Data collection

An anonymous patient database was employed, assembling core information from each clinical admission including details from the patient administration system, national hospital in-patient enquiry (HIPE) scheme, the patient electronic record, and laboratory data. HIPE is a national database of coded discharge summaries from acute public hospitals in Ireland [15]. The International Classification of Diseases, Ninth Revision, Clinical Modification (ICD-9-CM) has been used for both diagnosis and procedure coding from 1990 to 2005 and ICD-10-CM since then. Data included parameters such as the unique hospital number, admitting consultant, date of birth, gender, area of residence, principal and up to nine additional secondary diagnoses, principal and up to nine additional secondary procedures, and admission and discharge dates. Additional information cross-linked and automatically uploaded to the database includes physiological, haematological, and biochemical parameters.

### Risk predictors

Derangement of admission biochemical parameters may be utilised to predict clinical outcome. We have previously derived and applied an Acute Illness Severity Score (AISS) [16], predicting 30-day in-hospital mortality from parameters recorded in the ED. A weighted age adjusted score was derived; six risk groups (I–VI) were identified with cut-points for 30-day in-hospital mortality set at 1, 2, 4, 8, and 16%. We further adjusted for comorbidity as described below. In addition, categories of (1) no blood culture request, (2) negative blood culture, and (3) positive blood culture were identified and used as an adjustor in the multivariable logistic regression model [4].

### Comorbidity score

Patient morbidity was assessed by a Comorbidity Score [13]. To devise the score, we searched ICD codes that captured chronic physical or mental health disorders that limit people in activities that they generally would be expected to be able to perform were grouped according to the following ten systems: (i) cardiovascular, (ii) respiratory, (iii) neurological, (iv) gastrointestinal, (v) diabetes, (vi) renal, (vii) neoplastic disease, (viii) others (including rheumatological disabilities), (ix) ventilatory assistance required, and (x) transfusion requirement. In addition, we searched our hospital’s other databases for evidence of diabetes (Diamond database) [17], respiratory insufficiency (FEV1 < 2 L), troponin status (high sensitivity troponin ≥ 25 ng/L) [18], low albumin (< 35 G/dL), and anaemia (haemoglobin levels < 10 G/dL) or chronic renal insufficiency − MDRD < 60 mL/min * 1.73 m^2^ [19]. Each component of the score was then weighted according to 30-day in-hospital mortality.

### Statistical methods

Descriptive statistics were calculated for background demographic data, including means/standard deviations (SD), medians/inter-quartile ranges (IQR), or percentages. Comparisons between categorical variables and mortality were made using chi-square tests. We adjusted the outcome computation (30-day in-hospital mortality) for other known predictor variables including AISS [16], Comorbidity Score [13], and blood culture results [4]. Thirty-day in-hospital mortality is defined as a death occurring for any reason in the hospital within 30 days of the admission date. We employed a logistic model with robust estimate to allow for clustering; the correlation matrix thereby reflected the average discrete risk attributable to each of these predictor variables [16]. Of course, over a prolonged observation period of 6 years, many patients were admitted more than once. For example, over the totality of the database, those admitted more than once, twice, or three times was 49.5%, 31.8%, and 22.4%, respectively, with 5.4% admitted > 10 times each.

Logistic regression analysis identified potential mortality predictors and then tested those that proved to be significant univariate predictors (*p* < 0.1) by Wald test to ensure that the model included all variables with predictive power. We used the margins command in Stata to estimate and interpret adjusted subgroup predictions, controlling for other variables, using computations of average marginal effects. Margins are statistics calculated from predictions of a previously fitted model at fixed values of some covariates and averaging or otherwise over the remaining covariates.

The admitting service was regressed against the hospital length of stay (LOS), restricted to certain values, using truncated Poisson regression. We adjusted for AISS (8), Comorbidity Score (10), and blood culture status (9). We used robust standard errors for the parameter estimates, as recommended by Cameron and Trivedi [20]. The Poisson regression coefficients are the log of the rate ratio: one can interpret the coefficients in terms of incidence rate ratios (IRR).

Adjusted odds ratios (OR) and 95% confidence intervals (CI) were calculated for those predictors that significantly entered the model (*p* < 0.10). Statistical significance at *p* < 0.05 was assumed throughout. Stata v.17.1 (Stata Corporation, College Station, Texas) statistical software was used for analysis.

## Results

### Patient demographics

There were a total of 50,347 emergency medical admissions in 30,228 patients over the 6-year study period (2015–2020). The median (IQR) age was 70.1 (53.4, 81.7) years, with the upper 10% boundary at 87.9 years. The median (IQR) LOS was 7.7 (4.0, 15.9) days. In terms of overall requests, we compared patients referred to Acute Medicine (including Care of Elderly) or Specialty (Table 1). Those referred to Acute Medicine services were older at 72.9 years (57.0, 82.9) vs. 67.2 years (50.1, 80.2) with a similar LOS. There was some inequality in subgroup referral pattern with higher frequency of admissions under Acute Medicine in terms of higher Illness Severity (grades 4–6: 85.9% vs. 81.3%; *p* < 0.001), Charlson Index (> 0; 61.5% vs. 54.6%; *p* < 0.001), and Comorbidity Score (40.7% vs. 36.7%; *p* < 0.001). However, there was more frequent admissions under a Specialty in respect of suspected sepsis (blood culture request vs. no request) (24.4% vs. 27.0%; *p* < 0.001).

**Table 1:** Characteristics of emergency medical admissions by admitting service

	Acute Medicine	Specialty	*p*-value
	(*N* = 14,874)	(*N* = 15,984)	
Age (years)			
Mean (SD)	72.9 (57.0, 82.9)	67.2 (50.1, 80.2)	< 0.001
Median (Q1, Q3)			
Length of stay (days)	15.0 (23.30)	15.0 (22.98)	
Mean (SD)	7.7 (4.0, 15.7)	7.8 (3.9, 16.0)	0.95
Median (Q1, Q3)			
Gender	7395 (49.7%)	8332 (52.1%)	
Male	7476 (50.3%)	7649 (47.9%)	< 0.001
Female			
Hospital mortality	13,993 (94.1%)	15,070 (94.3%)	
Alive	878 (5.9%)	911 (5.7%)	0.445
Dead	72.9 (57.0, 82.9)	67.2 (50.1, 80.2)	
Acute Illness Severity Score			
1–3	2063 (14.1%)	2907 (18.7%)	< 0.001
4	2107 (14.4%)	2547 (16.4%)	
5	3057 (21.0%)	3073 (19.7%)	
6	7355 (50.4%)	7038 (45.2%)	
Comorbidity Score			
< 6	8819 (59.3%)	10,118 (63.3%)	< 0.001
6 < 10	5245 (35.3%)	5100 (31.9%)	
≥ 10	810 (5.4%)	766 (4.8%)	
Charlson Index			
0	4603 (32.4%)	4520 (29.8%)	< 0.001
1	4118 (29.0%)	3760 (24.8%)	
2			
Blood culture group	11,248 (75.6%)	11,665 (73.0%)	
1	3011 (20.2%)	3482 (21.8%)	< 0.001
2	612 (4.1%)	834 (5.2%)	
3	4603 (32.4%)	4520 (29.8)	

Includes all hospital deaths (after 30 days)

### Analysis of 30-day hospital mortality by admitting service

**Fig. 1:**
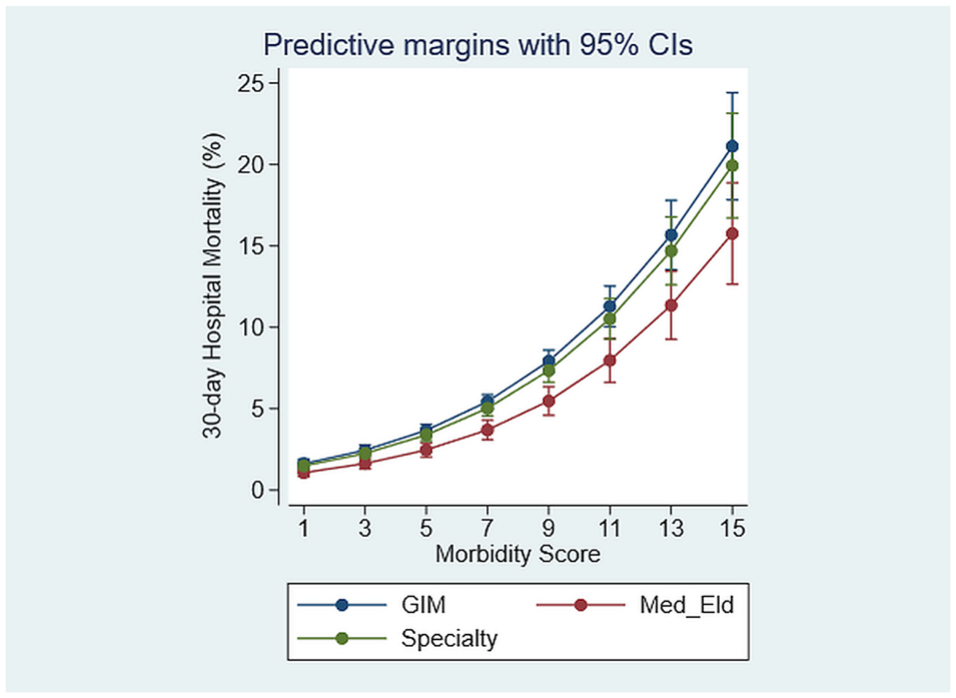
Thirty-day in-hospital mortality, increased as a curvilinear function of the underlying Comorbidity Score. The elderly on-take teams had a lower mortality (3.8%), compared with either the Acute Medicine (5.5%) or the specialists (4.8%)

The emergency medical admissions were admitted to firms classified as Acute Medicine (47.0%), a major medical subspecialty (36.1%), or Elderly Care (12.1%). The model adjusted 30-day in-hospital mortality rates for Acute Medicine 5.5% (95% CI: 5.1%, 5.9%) and/or for all Specialties 4.8% (95% CI: 4.4%, 5.3%) was not statistically different, whereas that for Elderly Care 3.8% (95% CI: 3.2%, 4.4%) was significantly lower (Fig. 1).

**Fig. 2:**
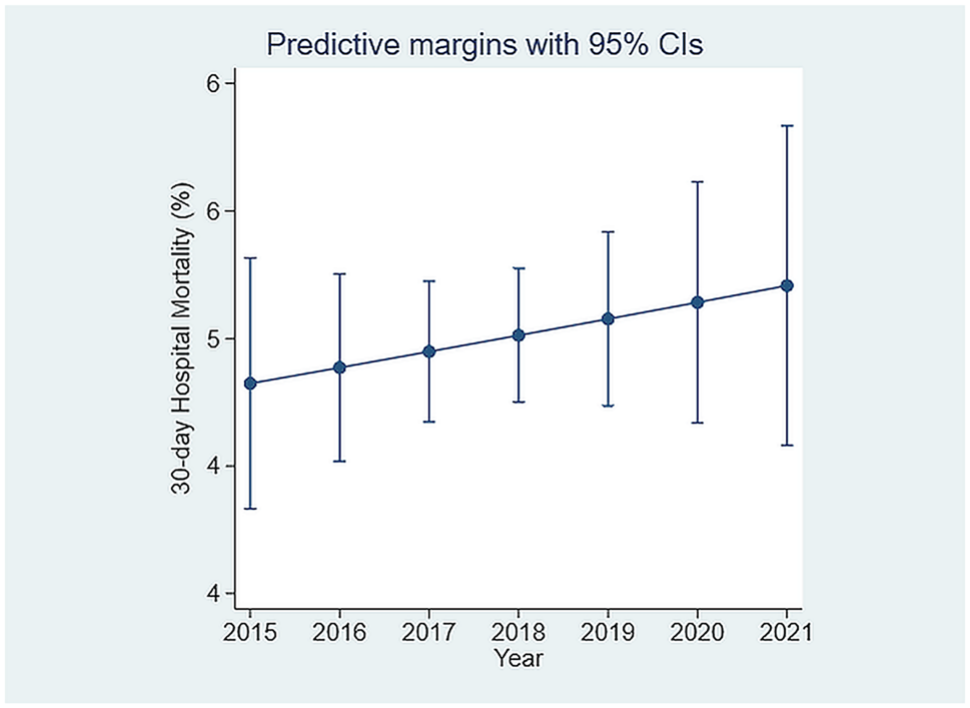
Thirty-day in-hospital mortality by year of admission. Compared with 2005, the trend of increasing mortality was sustained for each year from 2017 (*p* = 0.03) to 2020 (*p* < 0.001)

The data was adjusted for known predictor variables including AISS [16], Comorbidity Score [13], and blood culture results [4], all of which were highly significant, Table 2. It is clear, that in contrast to earlier trends from our AMAU, when year on year there were progressive falls in 30-day in-hospital mortality, this continuous improvement since 2003 has now ceased. There was a trend of increase in mortality year on year (Fig. 2), and comparing the 2015 nadir, the 30-day in-hospital mortality was significantly higher for each year after 2016 (one-way ANOVA; *p* < 0.02–*p* < 0.001).

**Table 2:** Logistic model of in-hospital 30-day mortality by admitting service

**Variable**	Odds ratio	Std. err	*z*	*p* > |*z*|	[95% Conf. interval]	
Acute Medicine	1.33	0.39	0.9	0.34	0.74	2.38
Elderly Care	0.81	0.25	−0.7	0.50	0.44	1.49
Specialties	1.18	0.35	0.6	0.58	0.66	2.12
Endocrinology	0.14	0.11	−2.5	0.01	0.03	0.64
Infectious disease	0.45	0.22	−1.6	0.11	0.17	1.19
Adjustors						
AHP referral	1.21	0.06	4.1	0.00	1.11	1.33
AISS	2.64	0.19	13.3	0.00	2.29	3.05
Sepsis	2.06	0.09	15.7	0.00	1.88	2.25
Charlson Index	1.24	0.05	5.3	0.00	1.15	1.35
Morbidity Score	1.22	0.02	15.0	0.00	1.19	1.25

### Truncated Poisson model of LOS by admitting service

There proved to be substantial differences in LOS, related to the admitting service, Table 3. The longest LOS was for a group of 16 specialties with 524 (1.7% of total) patients admitted via the ED as an emergency. These had a model predicted (adjusted) LOS of 11.1 days (95% CI: 10.4, 11.8). Shorter LOS were predicted particularly for Acute Medicine 8.7 days (95% CI: 8.6, 8.8) and Elderly Care 7.8 days (95% CI: 7.6, 8.0).

**Table 3:** Truncated Poisson model of admission LOS by admitting specialty

Variable	LOS	Std. err	*z*	*p* >|*z*|	[95% CI]	
Minor specialties	11.07	0.35	31.8	0.00	10.39	11.75
Acute Medicine	8.73	0.05	161.3	0.00	8.63	8.84
Elderly Care	7.78	0.10	75.5	0.00	7.57	7.98
Specialties	8.74	0.06	144.0	0.00	8.63	8.86
Endocrinology	10.73	0.32	33.9	0.00	10.11	11.35
Infectious disease	10.55	0.30	35.3	0.00	9.97	11.14

## Discussion

For many years following the implementation of an AMAU at St. James’s Hospital, there was an established pattern of year on year reduction in 30-day in-hospital mortality; we previously reported relative risk reduction of 31.9% by admission from 5.5 to 3.7% and when calculated per patient of 61.1% from 12.4 to 4.8% [21]. This fall in mortality over time was not a unique experience; Laudicella et al. [22] reported that UK mortality rates fell between 2003 and 2008 from 14.9 to 11.4%; our comparable per patient figures were from 12.3 to 9.5% over these same years. Aragon and Chalkley [23] estimated that admission mortality fell between 2002 and 2014 from 5.7 to 3.9%; our comparable admission statistics were from 5.5 to 3.8% over the same years.

Although the use of historical control data has limitations, the data suggests that the observed reduction in mortality over time was a general UK healthcare system finding and not specific to a particular structural or system delivery reform. Clearly, the reasons for such improvements may be systematic; AMAUs were also established in Australia and Ireland [24, 25], and the Hospitalist movement has been gaining in popularity in North America [26]. Other contributing factors may include restructuring of operational systems or improved patient flow [24, 25] together with the structural innovations due to the AMAU process [27–29] or even increased direct consultant participation at the coalface [30]. Notwithstanding the general benign background, progress is never linear or inevitable. The Irish economic collapse around 2008 resulting in the arrival of an IMF Troika that imposed swinging cutbacks; the HSE budgetary reduction from 15 to 12 billion annually was estimated at approximately 22% (14) and sustained for a decade. There is also the rising population trend of major obesity, with associated metabolic disease. The observed effect size, on 30-day hospital mortality, is only ~8%, but the sustained trend, with each year after 2016 statistically different from 2015, suggests an emerging pattern.

In a system improvement, as well as the general overall improved outcome parameters, one would anticipate that any major difference between ‘teams’ or specialist groups might be attenuated. We found no systematic difference between the overall 30-day in-hospital mortality between the various different elements of the admitting divisions. Of course, in any retrospective comparison, the major problem is inadvertent bias, introduced by selection, such as in specialty triage [24, 25] — colloquially termed ‘cherry-picking’. Unlike the randomised clinical trial where random allocation of patients to the different arms would be anticipated to secure comparability in baseline variables between the groups of interest, the ‘take’ system offers multiple points, where bias can be introduced. The only way to ensure a relative lack of bias in the ED is where the allocation is the receiving service is purely sequential, and this is largely not the case. This selection effect is one potential explanation for the discrepancies in admission profiles with patients admitted under Acute Medicine being older with more comorbidities and greater illness severity. Alternatively, this complex group of patients may be less likely to present with a clearly defined disease process and may be therefore rightly felt to be more appropriately admitted under a generalist who may have a greater skillset in navigating this complexity and differentiating the underlying disease mechanisms.

The bulk of the emergency case load fell on Acute Medicine (47.0%), and major subspecialties (36.1%); the contribution from Elderly Care (12.1%) was surprisingly modest, considering that 51% of the admissions were > 70 years. Of course, this may purely reflect the comparative consultant body numbers and relative service capacities. The 30-day in-hospital mortalities, for Acute Medicine, major medical specialties, and Elderly Care, were not different. The small number of admissions under endocrinology (*n* = 386) and infectious disease (*n* = 456) had much lower mortalities. In terms of hospital LOS, the minor specialties had a longer LOS than the mainstream; however, these represented 4.8% of all admissions. Elderly Care had a shortened LOS compared with either Acute Medicine or the major medical specialties; this could be due to better systems more attuned to problems of the elderly. These differences were not accounted for by a discrepancy in admission risk profile, related to the ‘on-call’ allocation.

Our original AMAU design, of two large contiguous wards of 60 beds, at the centre of the hospital was predicated on the principle of efficiency — there should ensure a reduction in-hospital LOS. Our course, as all the teams would be rounding in the same location, the ‘cross-talk’ might improve expertise applied on average to each case — with better outcomes. The experience was that the ‘cross-talk’ did work to boost expertise — there was a year on year improvement continuously from 2003 to 2018, with 30-day hospital mortality falling essentially as a linear function [14]. However, the anticipated LOS reduction did not in actuality occur — simply put asking for a ‘consult’ always takes time, from another busy service. Then the received advice/investigations have to be actioned.

Hospital LOS has received much scrutiny over time, due to the pressure on resources from increased demand the LOS has become a target, due to its direct association with hospital costs [1, 4, 6]. Studies have examined LOS predictors to inform intervention and targeting strategies [4, 5, 7–9]. A recent systematic review found that for patients with medically complex conditions, discharge planning, medication management, and interdisciplinary care teams were associated with inconsistent outcomes (LOS, readmissions, mortality) across populations [31]. There are no systematic reviews studying interventions for patients with socioeconomic risk; 70% of our admissions derive from a high deprivation inner-city population [32].

There is no single, agreed model of care across the health service; or how best to balance between the need for continuity of care or specialist input. Continuity of care, particularly at consultant level, is integral to the delivery of high quality care and maintaining patient flow [33]. However, there has been little critical evaluation of the two main suggested approaches of medical assessment and structuring; firstly concentrating on general wards with specialist input or alternatively directing appropriate patients to specialist wards as soon as possible, regardless of their expected length of stay [33]. Our AMAU opened in 2003 with the expectation that, in a major teaching hospital with high standards, we might achieve greater efficiencies (reduced LOS, readmission rates, and cost) at unchanged mortality outcomes. The model of care was a large 60-bedded Medical Admission Unit with rapid specialist support, but with the bulk of care delivery being via the ‘Physician of the day’. Over the subsequent > 18 years following its implementation, there was a RRR in 30-day in-hospital mortality by admission of 31.9% from 5.5 to 3.7% and by patient of 61.1% from 12.4 to 4.8%. The fall in mortality was not due to a change in admission policy or lower risk categories, but represented the outcome figure adjusted for major risk factors [16].

There are several limitations to the current study. Our institution covers an urban area and serves a population with high rates of intrinsic deprivation. It is possible that the results will not be replicated in other settings and populations and so further studies are needed to establish the consistency and external validity of our findings. In order to achieve sufficient power for our analysis, we have grouped various individual services with similar characteristics into our ‘specialty’ groups. It is possible that we are missing intra-specialty discrepancies which may be clinically important, this will require further larger studies to evaluate.

In conclusion, we have demonstrated no major differences in outcomes including mortality and LOS based on admitting specialty. There appeared to be unexpected discrepancies in risk profile and volume between the different specialty groups. The consistent annual trend towards improved mortality appears to have reversed.

## Data Availability

No further data is available for sharing. All pertinent data has been published in this manuscript.
